# Thrombospondin type-1 domain-containing 7A-associated membranous nephropathy after resection of rectal cancer: a case report

**DOI:** 10.1186/s12882-019-1236-y

**Published:** 2019-02-06

**Authors:** Shinya Taguchi, Yoshiki Koshikawa, Shoya Ohyama, Hiroko Miyachi, Hiroaki Ozawa, Hiroaki Asada

**Affiliations:** 1 Department of Nephrology, Okazaki City Hospital, 3-1 Goshoai Koryuzi-cho, Okazaki, Aichi 444-8553 Japan; 2 Department of Nephrology, Immunology and Vascular Medicine, Kidney Disease and Transplant Center, Shonan Kamakura General Hospital, 1370-1 Okamoto, Kamakura, Kanagawa 247-8553 Japan; 3 Department of Pathology, Okazaki City hospital, 3-1 Goshoai Koryuzi-cho, Okazaki, Aichi 444-8553 Japan

**Keywords:** Thrombospondin type-1 domain-containing 7A, Membranous nephropathy, Malignancy, Rectal cancer, Phospholipase A_2_ receptor

## Abstract

**Background:**

Thrombospondin type-1 domain-containing 7A (THSD7A) is a target antigen in idiopathic membranous nephropathy (MN). Patients with THSD7A-associated MN are known to have a high possibility of developing malignancy. However, there are few case reports on THSD7A-associated MN with malignancy, and details of its characteristics have not been clarified thoroughly. Here, we report the case of a 77-year-old male patient who was diagnosed with THSD7A-associated MN after resection of rectal cancer.

**Case presentation:**

A 77-year-old man who had developed bilateral peripheral edema, persistent proteinuria, and nephrotic syndrome was admitted to our hospital. He was diagnosed with MN based on a renal biopsy 3 years after resection of rectal cancer, and positive staining for THSD7A in both kidney and rectal cancer tissues suggested that these two diseases were related. Furthermore, THSD7A staining of metastatic lymph nodes revealed deposition of THSD7A in the secondary lymph follicles and sinus. Recurrence of rectal cancer was suspected; however, tumor recurrence was not observed on chest and abdominal computed tomography (CT) and colonoscopy. There was no lymph node enlargement. The patient was kept on observation with supportive therapy. Consequently, although nephrotic syndrome persisted, obvious recurrence and metastasis of the primary tumor were not observed.

**Conclusion:**

This is the first case in which pathological examination results suggested that THSD7A-associated MN was caused by rectal cancer. Based on the reports of THSD7A-associated MN with malignancy and the pathogenesis of MN, lymph node metastasis may be a risk for cancer-related MN.

## Background

Membranous nephropathy (MN) is a major cause of nephrotic syndrome in adults [1] and is a glomerular disease caused by the deposition of immune complexes on the epithelial side of the renal glomerular basement membrane [2]. Approximately 75% of MN is idiopathic, with secondary MN due to infection, malignancy, and autoimmune disease accounting for the rest [2]. With the discovery of phospholipase A_2_ receptor (PLA2R) in 2009 [3] and thrombospondin type-1 domain-containing 7A (THSD7A) in 2014 [4] as target antigens in idiopathic MN, the pathophysiology of MN is being investigated, and the diagnosis, treatment, and prediction of prognosis of MN are possible in the future [5–7]. THSD7A may be associated with MN and malignancy [8, 9], which was previously a cause of secondary MN [10, 11]. However, there are few case reports on THSD7A-associated MN with malignancy, and details on its characteristics have not been clarified. In this report, we present the case of THSD7A-associated MN after resection of rectal cancer.

## Case presentation

A 77-year-old man developed bilateral peripheral edema in August 2017. Persistent proteinuria and nephrotic syndrome were observed, and he was admitted to our hospital in September 2017. Past medical history included hypertension, cerebral hemorrhage, and rectal cancer. The rectal cancer was detected via a colonoscopy examination in July 2015, and a high anterior resection surgery with lymphadenectomy was performed in October 2015, with pathological diagnosis of rectal cancer, pT3N2bM0, pStage IIIC. Based on the sequelae of cerebral hemorrhage and performance status, no adjuvant chemotherapy was administered, and no recurrence was detected in follow-up. The patient’s family history was unremarkable. On admission, the blood pressure was 109/69 mmHg, the pulse was regular at 109 beats/min, and the body temperature was 36.8 °C. Physical examination revealed no abnormalities except for pitting edema of the limbs. Mild bilateral pleural effusion was confirmed by chest radiography. The size and the blood flow signal of both kidneys were normal in renal echography. Laboratory test results were as follows: total protein, 5.3 g/dL; albumin, 1.3 g/dL; serum creatinine, 1.07 mg/dL; total cholesterol, 293 mg/dL; glycosylated hemoglobin, 6.2%; white blood cell (WBC) count, 5000 cells/μL; hemoglobin, 12.3 g/dL; and platelet count, 23.7 × 10^4^/μL. The patient was positive for hepatitis C virus (HCV) antibody, but HCV RNA level was low. Tests for hepatitis B surface antigen, hepatitis B surface antibody, and human immunodeficiency virus antibody were negative. Urinalysis results were as follows: urinary protein excretion of 10.1 g/day, sediment containing 1–4 red blood cells, 1–4 WBCs per high-power field, 1–4 granular casts per whole field, and oval fat bodies. Further serological study results were as follows: IgG, 891 mg/dL; IgA, 176 mg/dL; IgM, 90 mg/dL; C3, 149 mg/dL; and C4, 41 mg/dL. Anti-nuclear antibody was present at 40-fold in a homogenous and speckled pattern, but the specific antibody was absent.

**Fig. 1:**
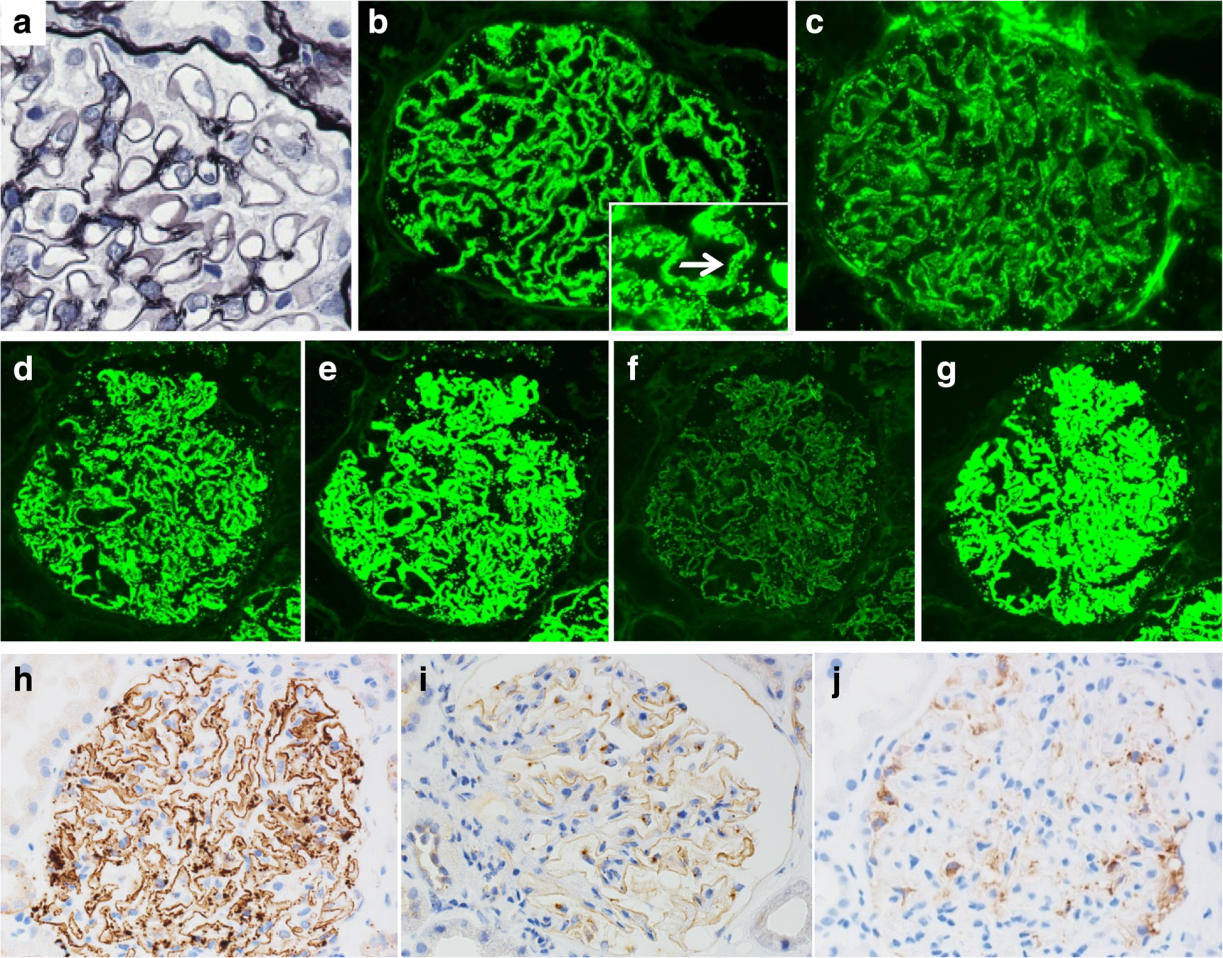
Microscopic findings of the glomeruli in the kidney. Periodic acid-methenamine silver staining shows no obvious abnormality (**a**). Immunofluorescence shows granular staining for IgG (**b**, arrow), C3 (**c**), IgG1 (**d**), IgG2 (**e**), IgG4 (**g**) along the capillary wall with weak staining for IgG3 (**f**). Immunohistochemical staining shows strong staining for THSD7A along the capillary wall in the case (**h**), in normal control (**i**), with negative staining for PLA2R in the case (**j**)

**Fig. 2:**
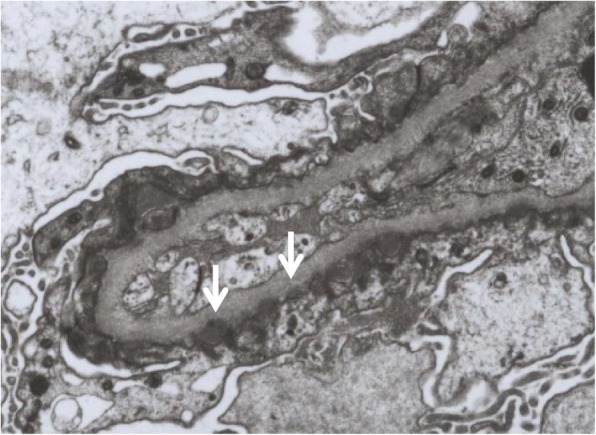
Electron microscopy shows subepithelial electron-dense deposits (arrows) (original magnification × 3000)

**Fig. 3:**
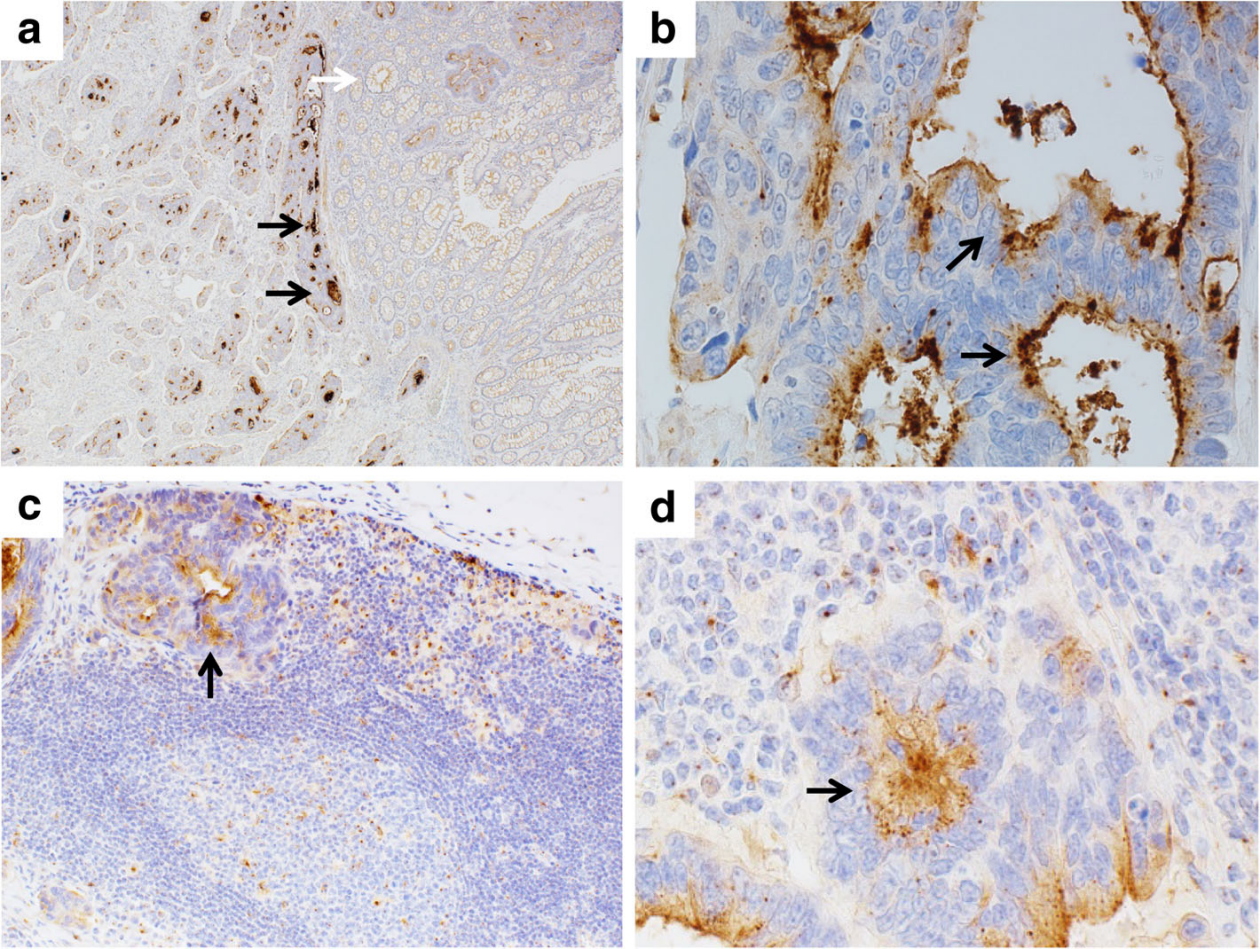
THSD7A staining in the rectal cancer, and metastatic lymph node. Strong staining for THSD7A is shown in tumor tissue (**a**, black arrows), with no staining in non-tumor tissue (**a**, white arrow). THSD7A shows luminal pattern staining in tumor tissue (**b**, arrows). THSD7A staining is shown in metastatic cell in lymph node (**c**, **d**, arrows). Original magnification **a**: × 40, **b**: × 600, **c**: × 200, **d**: × 600

A renal biopsy was performed the day after admission. Light microscopy (LM) showed that 9 of 42 glomeruli were globally sclerotic. Although the glomerular base membranes were thickened, spike formation was not observed (Fig. 1). Mild mesangial proliferation was observed in a few glomeruli. There was no endocapillary proliferation or crescent formation. Tubulointerstitial or vascular damage was absent. Immunofluorescence showed diffuse granular staining for IgG (2+), and C3 (2+) along the glomerular capillary walls (GCWs). Staining for IgA, IgM, and C1q was negative. Regarding the subclasses of IgG, strong staining was observed for IgG1, IgG2, and IgG4 and weak staining for IgG3 (Fig. 1). Electron microscopy showed subepithelial electron-dense deposits (Fig. 2). Consequently, a diagnosis of MN of Ehrenreich-Churg stage 1 was made. Additionally, we performed immunostaining for PLA2R and THSD7A of the glomeruli and rectal cancer tissues with metastatic lymph nodes to discriminate between idiopathic and secondary MN (Fig. 3). In the glomeruli, granular staining for THSD7A along the GCW was observed with negative staining for PLA2R. In the rectal cancer tissue, THSD7A staining was positive, showing a mainly luminal pattern, which was reported as the staining pattern of rectal cancer [12]. THSD7A staining of metastatic lymph nodes revealed deposition in the secondary lymph follicles and in the sinus. Based on the above findings, a diagnosis of THSD7A-associated MN was made, which was associated with rectal cancer. We suspected the recurrence of rectal cancer; however, there was no tumor recurrence on chest and abdominal CT examination and colonoscopy. No lymph node enlargement was observed.

Treatment of tumors cures tumor-associated MN [13], but the patient did not want chemotherapy, and therefore he was kept on observation with supportive therapy. Consequently, nephrotic syndrome still persists, but obvious recurrence and metastasis of the primary tumor have not been observed.

## Discussion and conclusions

In this case, we encountered THSD7A-associated MN, which may have been caused by rectal cancer based on pathological examination results. To date, there have been reports of cases of THSD7A-associated MN with gallbladder cancer [8] and endometrial cancer [9]. However, our case report is the first to show an association of THSD7A-associated MN with rectal cancer by pathological examination. All three cases [(1) our case, (2) MN with gallbladder cancer, and (3) MN with endometrial cancer as described above], there was lymph node metastasis. Considering the assumed mechanism of the development of MN, lymph node metastasis might be a possible risk of MN associated with malignancy.

This case suggested that secondary MN because of rectal cancer may occur via THSD7A. Megalin was identified as the first responsible antigen for MN based on a study of Heymann nephritis, which is the rat experimental model for MN [14]. However, megalin is mainly present at the proximal tubular brush border, and not in glomerular epithelial cells. Therefore, megalin may not be an antigen related to human MN [15]. Subsequently, neutral endopeptidase was reported as a responsible antigen for human MN from a study of nephrotic syndrome in neonates [16], but it was unable to explain the onset of adult MN. In 2009, PLA2R, a protein expressed in glomerular podocytes, was identified as the responsible antigen for idiopathic MN, and it was found that anti-PLA2R antibody was detected in about 70% of idiopathic MN [3]. In 2014, THSD7A was identified as the second responsible antigen after PLA2R [4]. THSD7A has been detected in about 2–9% of idiopathic MN [4, 17, 18], whereas 20% of patients with THSD7A-associated MN had malignant tumors found within 3 months from the diagnosis of MN [9]. This suggested that THSD7A may be the responsible antigen for malignancy-associated MN, which was previously known as one of the causes of secondary MN [10, 11]. THSD7A, a transmembrane receptor similar to PLA2R, is related to angiogenesis by regulating migration of vascular endothelial cells and activates focal adhesion kinase [19, 20], which is a protein tyrosine kinase that plays an important role in cell proliferation, differentiation, and apoptosis and may be expressed in various tumors and is related to proliferation and metastasis [21, 22]. Stahl et al. reported that THSD7A was expressed in different staining patterns in various tumors, and that 43% of colorectal cancer tissues were THSD7A-positive in a mainly luminal pattern, whereas normal colorectal tissues exhibited a cytoplasmic pattern [12]. In this case, too, strong staining of THSD7A was observed in a luminal pattern in rectal cancer tissue [12]. Hoxha et al. reported 8 cases of THSD7A-associated MN with malignancy, among which 2 cases were diagnosed by THSD7A-positive staining in both kidney and tumor tissues from gallbladder and endometrial cancers [9]. To the best of our knowledge, this is the first case of THSD7A-related MN associated with rectal cancer, diagnosed by THSD7A-positive staining in both kidney and tumor tissues.

Tumor metastasis, particularly lymph node metastasis, might be a risk factor for cancer-related MN. In this case and in 2 other reported cases of THSD7A-associated MN with malignancy, there was lymph node metastasis, and in both those patients THSD7A was positive in metastatic lymph nodes [8, 9]. Considering that the follicular dendritic cells in the lymph nodes are a type of antigen presenting cells and play an important role in antigen-specific antibody production, it is suggested that anti-THSD7A antibody produced by recognizing THSD7A as a tumor antigen in the lymph node binds to THSD7A expressed in the glomerular podocytes and forms an immune complex and leads to MN development [8]. These findings also suggested that antigen recognition might happen in the lymph node, and lymph node metastasis might be a risk factor for cancer-related MN. These are merely inferences, and it is necessary to investigate the THSD7A staining of tumor tissues and lymph nodes in patients without lymph node metastasis.

Regarding the pathogenesis of MN, it is suggested that idiopathic MN develops based on the concept of in situ immune complex formation, in which the antigens responsible are present in glomerular podocytes and immune complexes are formed in situ [23]. Moreover, the discovery of PLA2R and THSD7A supports this hypothesis [3, 4]. In cancer-related MN, it is assumed that antibodies against tumor antigens are produced and form immune complexes with similar structural endogenous antigens on the podocytes in situ, or immune complexes formed in the circulation are trapped in the capillary wall, but the details are unclear [24]. In THSD7A-associated MN, it is suggested that an antibody against THSD7A, a tumor antigen, is produced and forms an immune complex in situ with THSD7A as an endogenous antigen in podocytes, resulting in the development of MN [8]. As it became clear that THSD7A-associated MN and tumor are related, the concept of in situ immune complex formation might be the major pathological condition in tumor-associated MN as with idiopathic MN. In this case, although there is no obvious recurrence, there is a possibility that lymph node metastasis may remain because postoperative chemotherapy has not been performed. Antibodies against the THSD7A antigen expressed in the remaining tumor may have been produced, which may have led to MN.

The association between malignancy and MN has been reported many times since a report by Lee et al. [10] and a meta-analysis reported that the prevalence of malignancy in patients with MN was 10%, the majority of the cancers were lung cancer and prostate cancer, and 20% of patients had a tumor before the diagnosis of MN [11]. If antibody production associated with recognition of tumor antigen is considered as the origin of MN, tumors will develop prior to the onset of MN, but whether the tumor or MN is diagnosed first depends on the tumor site and MN activity. Because the outcome of the tumor strongly affects the prognosis, it is clinically important to screen carefully for tumors, and even if they cannot be found, to continue careful follow-up for tumor development when the patient has findings of negative PLA2R [25], positive IgG1 and IgG2, and negative IgG4 in the staining for IgG subclasses [26, 27] that are all reported to increase tumor risk, and positive THSD7A. There was also a case reported about recurrence of endometrial cancer following the diagnosis of MN 17 years after curative surgery [5], suggesting that recurrence of cancer might cause MN in some cases. In our case, MN developed three years after resection of rectal cancer and regional lymph nodes by surgery; recurrence of rectal cancer could occur in the future, as in the case report above, and careful follow-up is therefore necessary.

There have been several prognostic criteria proposed for tumor-associated MN, according to which there should be a temporal association between malignancy and MN and clinical and pathologic remission of MN due to treatment of the tumor [28]. In this case, unlike the two cases above, rectal cancer and MN are not necessarily related to each other because there has been no treatment for rectal cancer. Furthermore, as the patient is elderly, and it is possible that development of rectal cancer is just coincidental. However, as the patient’s findings were negative for PLA2R staining and positive for IgG1 and IgG2 staining in glomeruli, this diagnosis is likely to be secondary MN. Furthermore, THSD7A-positive staining in both the kidney and tumor tissues suggests that the association between rectal cancer and MN is highly certain.

We encountered a case of THSD7A-associated MN, which was believed to have been caused by rectal cancer. Per our literature search, this is the first case of THSD7A-associated MN with rectal cancer diagnosed by THSD7A-positive staining in both kidney and tumor tissues. It was suggested that tumor metastasis, particularly lymph node metastasis, might be a risk factor for the onset of cancer-related MN. The pathophysiology of cancer-related MN, which has not been clarified until now, is being investigated due to the discovery of the association between THSD7A-associated MN and malignancy, but many aspects are still unclear, such as the pathophysiology of cancer-related MN with negative-THSD7A staining, the site of antigen recognition, and the risk factors for cancer-related MN. It is presumed that there are a few cases in which malignancy and idiopathic MN have coincidentally developed among cases diagnosed as secondary MN due to malignancy; however, it is expected that these will be distinguished by making use of the staining for PLA2R, IgG subclasses, and THSD7A, and that findings of cancer-related MN will be accumulated in the future.

## Abbreviations

CT: Computed tomography
GCWs: Glomerular capillary walls
HCV: Hepatitis C virus
MN: Membranous nephropathy
PLA2R: Phospholipase A2 receptor
THSD7A: Thrombospondin type-1 domain-containing 7A
WBC: White blood cell
